# Formulation and Nanotechnology-Based Approaches for Solubility and Bioavailability Enhancement of Zerumbone

**DOI:** 10.3390/medicina56110557

**Published:** 2020-10-23

**Authors:** Siddharth S. Kesharwani, G. Jayarama Bhat

**Affiliations:** 1 College of Pharmacy, Roseman University of Health Sciences, South Jordan, UT 84096, USA; 2 Department of Pharmaceutical Sciences, College of Pharmacy and Allied Health Professions, South Dakota State University, Brookings, SD 57007, USA; jayarama.gunaje@sdstate.edu

**Keywords:** zerumbone, natural products, formulation development, drug delivery systems, pharmacological effects of zerumbone, and molecular targets of zerumbone

## Abstract

About 40–70% of drug molecules in the clinical development pipeline suffer from one of either low aqueous solubility, poor absorption, or extremely low bioavailability. Approximately 75% of the world population relies on traditional therapies and therefore there has been a growing interest in the utilization of natural compounds. Zerumbone is one such natural compound, classified as a sesquiterpenoid that is extracted from the essential volatile oils of rhizomes from *Zingiber zerumbet*. It possesses strong antitumor, antioxidant, antimicrobial, and anti-inflammatory activity. However, despite promising preclinical studies demonstrating the therapeutic utility of zerumbone, its clinical development has been limited due to its low aqueous solubility, poor absorption, or associated low bioavailability. Multiple reviews demonstrating the pharmacological effects of zerumbone for various diseases have been published. However, to our knowledge, no review demonstrates the various formulation strategies developed to overcome the biopharmaceutical challenges of zerumbone. The purpose of this review is to provide a comprehensive perspective on zerumbone as a molecule for formulation development. A section related to pharmacokinetics, toxicity, and patents of zerumbone is included. This review provides the importance of developing novel formulations of zerumbone to overcome its biopharmaceutical challenges thereby advance its potential in the treatment of various diseases.

## 1. Introduction

With the development of novel drug discovery and development methodologies, there has been an increase in the number of new drug candidates in clinical development [1]. It estimated that 40–70% of compounds in clinical drug development suffer from low oral bioavailability due to poor aqueous solubility and absorption [2,3]. Drug solubility and permeability are the two fundamental parameters that control the rate and extent of drug absorption. The preclinical and clinical assessment of new drug candidates is hampered because of these challenges mentioned above [4]. Poorly soluble and absorbable drugs pose a significant challenge to the formulation scientists to develop a suitable dosage form that can enhance their solubility and bioavailability [5,6,7,8]. The solubility and bioavailability of a drug can be improved through multiple approaches [9] such as chemical modification, salt formation, amorphization, particle size reduction [10], or through different formulation development strategies [11] such as solid dispersions [12], molecular complexation [13,14,15], lipid-based formulations [16,17,18,19], micelles [20,21], and nanoparticles [22]. 

About 70–80% of the world population relies on traditional therapies, most commonly involving natural compounds [23,24]. In the recent years, there has been considerable attention on natural compounds which is primarily based on the premise that these compounds can promote health and alleviate diseases [23,25]. Approximately 20–30% of the modern drugs that are prescribed worldwide are of plant-origin. Most well-known natural compounds are polyphenols, flavonoids, and terpenes [26]. Zerumbone is one such natural compound, classified as a sesquiterpenoid that is extracted from the essential volatile oil of rhizomes from wild ginger, *Zingiber zerumbet* [27,28,29,30,31,32,33,34,35,36,37,38]. Zerumbone has been shown to possess strong antitumor [27,30,39], antioxidant [40], antimicrobial [41], anti-nociceptive [42], hepatoprotective [43], immunomodulatory [44], anti-inflammatory [33,45,46], and gastro-protective [40] activity. However, despite promising in-vitro and in-vivo studies demonstrating the therapeutic utility of zerumbone, its preclinical and clinical development has been limited due to its poor water solubility, poor absorption, or low bioavailability, limiting its targeting into various tissues and organs of interest. Hence, to advance the therapeutic potential in various diseases and overcome the biopharmaceutical challenges of zerumbone, it is essential to develop advanced formulation and nanotechnology-based approaches [28,35,36,37,47,48].

Multiple reviews demonstrate the beneficial pharmacological effects of zerumbone for various diseases such as different types of cancer, wound healing, obesity, inflammatory bowel diseases [27,28,29,30,31,32,33,34,35,36,37,38,49]. However, to our knowledge, no review demonstrates the various formulation strategies developed to overcome the biopharmaceutical challenges related to zerumbone. The purpose of this review is to provide a comprehensive perspective on zerumbone as a candidate molecule for formulation development. The source, physicochemical properties, structure, and molecular targets of zerumbone are covered in brief details. Various formulation development strategies to overcome the biopharmaceutical challenges have been discussed in sufficient details. We have also reviewed studies related to pharmacokinetics, toxicity, and patents of zerumbone. We believe that this review will provide the importance of developing novel formulations of zerumbone to overcome its biopharmaceutical challenges and to advance its potential as a therapeutic agent in the treatment of various diseases.

## 2. Source, Chemistry, Physicochemical Properties, and Pharmacological Effects of Zerumbone

**Figure 1:**
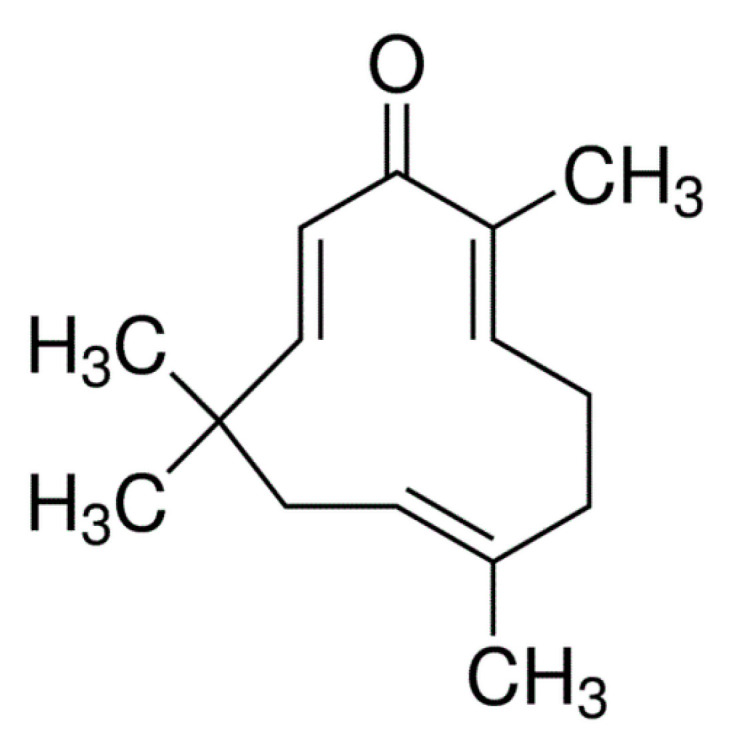
Chemical structure of zerumbone [29,56].

Zerumbone (2E, 6E, 10E)-2,6,9,9-tetramethylcycloundeca-2,6,10-trien-1-one is a natural monocyclic eleven membered sesquiterpene (three isoprene units) isolated from the rhizomes of the wild ginger *Zingiber zerumbet* [50,51]. Zerumbone is isolated from Zingiberaceae species, especially Zingiber and Curcuma species. It contains α, β-carbonyl-based moiety with three double bonds [52,53,54,55]. Research from studies has shown that ~12–73% of Zerumbone in *Zingiber zerumbet* is in the rhizome oils [56,57]. Zerumbone is also known as a pinecone, asian, wild, or shampoo ginger. The molecular formula of zerumbone is C_15_H_22_O [33]. It is completely soluble in dimethyl sulfoxide (DMSO) and ethanol but has extremely poor solubility in water (~1–1.5 mg/L at 25 °C) [34,58]. Zerumbone is highly lipophilic and the poor water solubility contributes to poor absorption, low oral bioavailability, and limited targeting to tissues and organs of interest [28,35,36,37,47,48]. The chemical structure and other physicochemical characteristics of zerumbone are presented in Figure 1 and Table 1, respectively.

**Figure 2:**
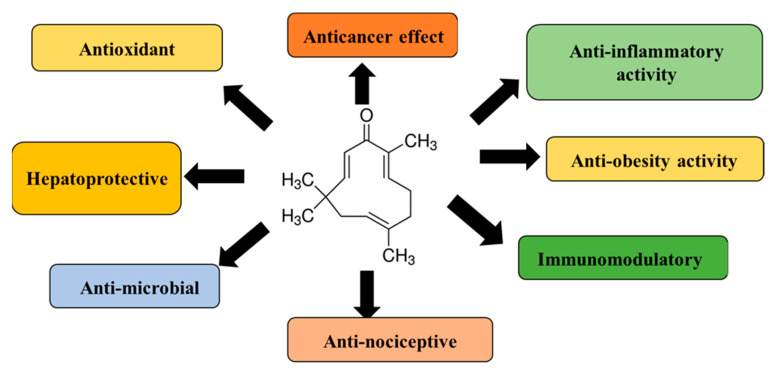
Summary of pharmacological effects of zerumbone.

**Figure 3:**
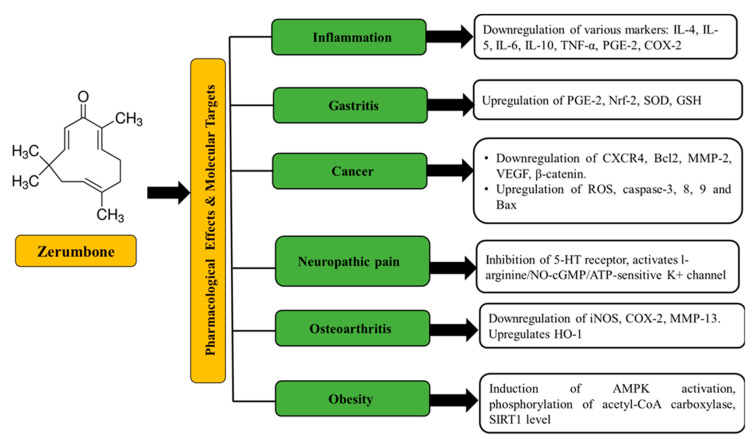
Molecular targets of zerumbone for various diseases [49].

Zerumbone is known to exhibit numerous biological and pharmacological effects (Figure 2) such as antitumor, antioxidant, antimicrobial, anti-nociceptive, hepatoprotective, immunomodulatory, anti-inflammatory, gastro-protective, and antiproliferative through modulation of various molecular targets (Figure 3) and signaling mechanisms. Multiple reviews and extensive amount of studies have been performed detailing the molecular targets of zerumbone for various chronic diseases and have been reviewed in references elsewhere in great detail [27,28,30,31,32,33,34,35,36,37,38,39,43,45,46,47,49,56,59,60].

## 3. Formulation Development and Drug Delivery Strategies of Zerumbone

With the advancement of combinatorial chemistry, high-throughput screening and cell-based assays, new chemical entities (NCEs) are being developed. However, nearly 40% of the drugs currently in the clinical drug development pipeline and ~75% of therapeutic molecules in the market suffer from low aqueous solubility and poor oral bioavailability. These biopharmaceutical limitations affect the discovery stage studies and thereby lead to a significant delay in the development of new drugs [3,4,9,12]. According to the biopharmaceutical classification system (BCS), the drug dissolution profile and solubility are major factors that affect gastrointestinal permeability, bioavailability, and clinical response. Drugs with poor aqueous solubility suffer from low bioavailability and limited transport after oral administration [1,2,3]. Thus, it is imperative to develop strategies to overcome the solubility and bioavailability concerns of poorly soluble compounds. To overcome these biopharmaceutical challenges, numerous strategies have been applied, such as chemical/structural modification, chelation, bio-conjugation, using a water-soluble polymer, inclusion complex formation, amorphous solid dispersions, reduction of particle size, lipid-based technology, nanocrystal technology, and nanoparticulate technology.

**Figure 4:**
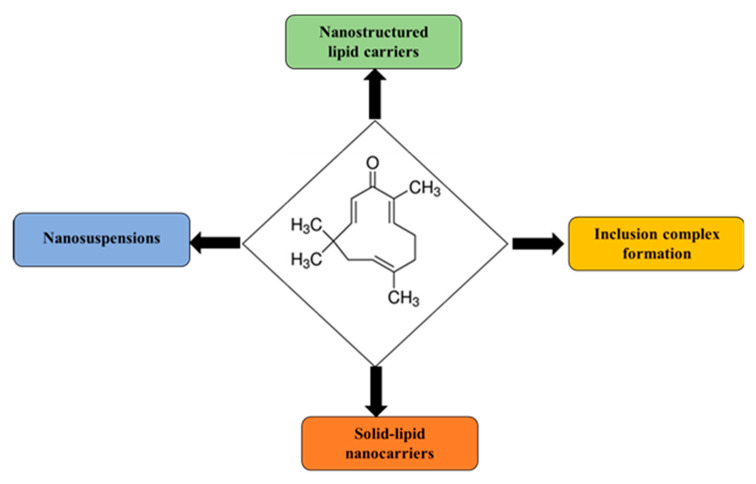
Formulation strategies employed to improve the solubility and bioavailability of zerumbone.

Zerumbone possesses numerous beneficial pharmacological activities as outlined above [49]. Despite having promising pharmacological activities, the preclinical and clinical utility of zerumbone has been limited due to its low aqueous solubility and poor oral bioavailability. The subsequent section and the Table 2 provides a concise review of the formulation development strategies that have been employed for the delivery of zerumbone. It provides details about the type of formulation developed, components of the delivery system, method of preparation, purpose, therapeutic indication, and the major results. The purpose is to provide researchers with the idea of the studies that have been published and the major area to concentrate upon when developing novel drug delivery strategies for zerumbone with the overall goal to enhance its therapeutic potential. Thee major approaches have been explored and they include: (i) nanostructured lipid carriers, (ii) inclusion complexes of cyclodextrins, and (iii) nanosuspensions (Figure 4). The majority of the studies demonstrate an enhancement of in-vitro aqueous solubility, stability, and in-vitro cytotoxicity of zerumbone-based formulations in cancer cell lines. However, none of these drug delivery strategies study the in-vivo pharmacokinetics or evaluate the in-vivo pharmacological activity of zerumbone.

### 3.1. Nanostructured Lipid Carriers

Lipid nanocarriers, include solid lipid nanoparticles (SLNs), and nanostructured lipid carriers (NLCs), are recognized as suitable carriers for drug delivery to enhance the solubility of poorly water-soluble compounds [19]. SLNs are the first type of lipid carrier to consist of solid and biocompatible matrixes [61]. Nanostructure lipid carriers contain both solid and liquid lipids. Specifically, the hybrid lipid scheme has been studied to ensure excellent biological applicability, controlled drug release, and possibilities of large industrial development in comparison with SLNs. NLCs are a modified form of SLNs which provide enhanced drug stability, controlled and site-specific drug release, enhanced drug loading capacity, and prevention of drug expulsion during storage [17,18,62]. The NLCs surface can be modified and loaded with hydrophobic or hydrophilic drugs. The carrier material is biodegradable and safe when administered in-vivo with enhanced physical and chemical stability [16,63]. 

Multiple zerumbone-loaded nanostructured lipid carriers have been formulated. Rahman et al. developed nanostructured lipid nanocarriers of zerumbone using hot, high-pressure homogenization technique and evaluated the antileukemic effect. Through a thorough physio-chemical and in-vitro biological characterization, the authors report stability, sustained release characteristics, and high cytotoxicity of zerumbone-loaded lipid nanocarriers in human lymphocytic leukemia cells as compared to zerumbone [36]. In another study, Rahman et al. extended the previous study to evaluate the acute toxicity of zerumbone-loaded nanocarriers in BALB/c mice after oral administration. The author’s report 50% lethal dose (LD_50_) of zerumbone-loaded nanocarriers to be higher than 200 mg/kg, and that this is safe via the oral route of administration [37,56]. In separate studies by Nathaniel et al. [35] and Foong et al. [28] evaluated the ability of zerumbone-loaded nanocarriers to induce apoptosis in human colorectal cancer cell lines and canine mammary adenocarcinoma cells (Table 2).

### 3.2. Inclusion Complexes of Cyclodextrins

Cyclodextrins are cyclic oligosaccharides constituted by six (α-cyclodextrin), seven (β-cyclodextrin), and eight (γ-cyclodextrin) glucopyranose units linked by α-(1, 4) bonds. Hydroxypropyl-β-cyclodextrin (HP-β-CD) is cyclodextrin derivative that has been studied widely in the field for drug encapsulation because of its ability to form inclusion complexes and the ability to increase aqueous solubility [64,65,66,67,68]. In a study by Eid et al., the authors evaluated the inclusion complex formation of HP-β-CD and zerumbone in an aqueous medium to enhance the aqueous solubility of zerumbone. Through a combination of multiple physicochemical characterization methods, the authors report the formation of inclusion complexes of zerumbone and HP-β-CD leading to an increase in solubility, stability, and bioavailability. This study further enhanced the potential of zerumbone as a therapeutic molecule via oral administration [60].

### 3.3. Nanosuspensions

Formulating nanosuspensions is one of the strategies to improve the oral bioavailability of poorly soluble drugs. Since the introduction of the first nanosuspension in 1992, the 1^st^ product to be approved by the Food and Drug Administration (US-FDA) in 2000 was Rapamune^®^, an immunosuppressive agent. Currently, there are more than 15+ products in the market, which are growing fast, due to the potential of this technology. The size of the nanosuspensions improves the saturation solubility and dissolution rate of the drug, thereby leading to an increase in oral bioavailability. In addition, nanosuspensions also provide enhanced stability, reduced toxicity, and increased pharmacological activity [69,70,71,72,73]. With the purpose to increase the aqueous solubility of zerumbone, Shadab et al. formulated nanosuspensions of zerumbone using high-pressure homogenization (HPH) with sodium dodecyl sulfate (SDS) and hydroxypropyl methylcellulose (HPMC) as stabilizers. The optimized formulation was characterized by various physicochemical properties. The authors report an increase in solubility, stability, and dissolution profile of zerumbone nanosuspension as compared to zerumbone alone. The results from this study provide evidence of the potential of nanosuspension technology to enhance the therapeutic potential of zerumbone in the future and warrant further in-vivo evaluation [48].

## 4. Pharmacokinetics and Toxicity of Zerumbone and Zerumbone Formulations

Zerumbone has been found to possess multiple beneficial pharmacological effects as outlined above. Despite the plethora of studies demonstrating the activities of zerumbone, there exists only limited evidence regarding the pharmacokinetics and toxicity of zerumbone [31,32,37,59,60]. Based on the potential therapeutic utility of zerumbone, it is expected that zerumbone would be used over a prolonged time and it is essential to evaluate the pharmacokinetics and toxic effects of the compound in both acute and chronic settings [74]. The subsequent section reports the limited preliminary investigations regarding the in-vitro and in-vivo toxic effects of zerumbone. Most of these studies are performed from three perspectives, mainly acute/chronic toxicity, genotoxicity, and cytotoxicity examinations. More preclinical and clinical toxicity studies are warranted to establish zerumbone as a therapeutic drug for the prevention and treatment of various chronic diseases. Furthermore, it is also imperative to study the pharmacokinetics of zerumbone and zerumbone formulations to aid in the development of this compound and overcome the solubility and associated low bioavailability concerns. All the relevant studies performed so far have been briefly explained and summarized in the subsequent section and Table 3.

Rahman et al. studied the acute toxicity of zerumbone and zerumbone-loaded nanocarriers on the BALB/c mice model for 14 days via oral route of administration. They evaluated animals for clinical and behavioral abnormalities, toxicological symptoms, feed consumption, gross appearance, serum biochemical parameters, total hemogram, and bone marrow stem cells. The authors report that all the treated mice and their tissues (liver, kidney, spleen, lung, heart, and brain tissues), serum biochemical parameters, total hemogram, and bone marrow were normal. In addition, the study reports that 100 and 200 mg/kg zerumbone-loaded nanocarriers did not show any signs of toxicity or mortality in BALB/c mice. Overall, it was found that both zerumbone and zerumbone-loaded lipid nanocarriers are potentially safe to administer via the oral route [37].

Jin et al. evaluated the single and repeat-dose toxicity of zerumbone in mice. The authors evaluated body and organ weight, food and water consumption, hematology, and serum biochemistry, as well as histology effects. The study reports that there were no significant differences in the general condition, serum biochemistry, growth, organ weights, hematology, or histopathological analysis in the repeat dose toxicity. Overall, it can be concluded that zerumbone is safe to administer. Zubairi et al. studied the in-vitro and in-vivo genotoxic effects of zerumbone and reported that higher doses (1000 mg/kg body weight) of zerumbone could potentially be genotoxic and cytotoxic [32].

Ibrahim et al. evaluated the acute toxicity and the effect of a single dose of zerumbone on the kidney and liver functions in Sprague Dawley rats. The overall goal was to determine the median lethal dose (LD_50_) of zerumbone and the effect of three different doses of zerumbone below the LD_50_ on various parameters such as blood biochemistry, hepatic, renal histopathologies, lipid peroxidation in rats. The results from this study report the LD_50_ of zerumbone to be 1.84 gm/kg. It was also found that zerumbone at 100–200 mg/kg had no toxic effects on the rats under the study. However, doses of 500mg/kg of zerumbone induced hepatocellular and nephrocellular damage leading to kidney and liver failure [31]. 

Eid et al. studied the pharmacokinetics of zerumbone-based inclusion complexes of hydroxypropyl-β-cyclodextrin (HP-β-CD) via intravenous and intraperitoneal administration. The authors report various pharmacokinetic parameters of zerumbone and zerumbone-based inclusion complexes of cyclodextrin. It was found that the HPβCD had a negligible effect on the pharmacokinetic parameters of zerumbone [60].

## 5. Patents Related to Zerumbone and Zerumbone Formulations

An extensive search for zerumbone and its formulations was conducted using Google Patents (patents.google.com), USPTO Patent full-text search (www.uspto.gov), WIPO IP Portal’s Patent scope (https://patentscope.wipo.int/), and Espacenet (worldwide.espacenet.com/patent/). After a careful review, it was found that only a few patents were directly related to zerumbone and its formulations. Table 4 provides the details of the patented systems and their clinical application. From Table 4, it is clear that very few patents exist in the area of formulations of zerumbone and there exists an enormous opportunity for formulation scientists to develop novel formulations. In addition, most of these patented systems are related to a particular formulation type with utility for a specific disease thereby being very narrow in its claims and utility. This further provides the opportunity to expand the scope and develop novel technologies related to zerumbone with broader applicability.

## 6. Conclusions and Future Outlook

Drugs with poor solubility suffer from poor oral bioavailability and limited transport after oral administration. Zerumbone possesses numerous beneficial pharmacological activities as outlined above. Despite having promising pharmacological activities, the preclinical and clinical utility of zerumbone has been limited due to its poor aqueous solubility, poor absorption, and low oral bioavailability. The formulation development studies performed are limited to only in-vitro or preliminary in-vivo studies. New formulations and delivery methods are needed to increase the solubility of poorly soluble drugs, including zerumbone. The studies on formulation development are limited to nanostructured lipid carriers, nanosuspensions, gels, and inclusion complex formation with cyclodextrins. To our knowledge, no data have been reported for its pharmacokinetics and pharmacodynamics in large animals and humans. For the successful development of water-soluble and stable formulation, it is important to carry out further investigations using various tests (toxicity, pharmacokinetics, and pharmacological studies) under acute and chronic setting. Furthermore, it is essential to perform a thorough physicochemical and biopharmaceutical characterization to translate the potential of zerumbone as a therapeutic agent. The findings from all the researches reviewed in this paper provides conclusive evidence that zerumbone has a strong potential for formulation development for various diseases. In addition, there is a need to conduct animal studies, pharmacokinetic and pharmacodynamics evaluation, detailed toxicological, and human clinical trials to ascertain the efficacy, usefulness, and safety of zerumbone.

## Figures and Tables

**Table 1:** Physicochemical characteristics of zerumbone [56].

Characteristics	Description
Occurrence	Zingiber species
Chemical class	Sesquiterpene
IUPAC name	(2E, 6E, 10E)-2,6,9,9-tetramethylcycloundeca-2,6,10-trien-1-one
Molecular formula	C_15_H_22_O
Molecular weight	218.3 g/mol
Lop P	3.9
Chemical structure	Three-double bond (two conjugated and one isolated), α, β-unsaturated carbonyl group, and a double conjugated carbonyl group in an 11-membered ring structure
Flashing point	272°F
Boiling point	321–322 °C at 760 mmHg
Melting point	65.3 °C
Appearance	solid white crystals or powder
Stability	Stable for at least 2 years when stored at −20 °C
Solubility	Freely soluble in organic solvents such as ethanol and dimethyl sulfoxide (DMSO)Solubility in water ~1–1.5 mg/L at 25 °C

**Table 2:** Formulation development strategies and delivery systems of zerumbone.

Nr	Formulation Strategy	Method of Preparation	Components of Delivery System	Therapeutic Indication	Purpose	Major Results/Conclusions
1	Nanostructured lipid carriers [36]	Hot, high-pressure homogenization	**Lipid dispersion:** hydrogenated palm oil, olive oil, and lipoid S100 **Aqueous dispersion:** Sorbitol, Tween-80 and thiomersal	Leukemia	Treatment of leukemia	The zerumbone-loaded nanocarriers depict sustained release characteristics and high cytotoxicity in human T-cell acute lymphocytic leukemia.
2	Nanostructured lipid carriers [37]	Hot, high-pressure homogenization	hydrogenated palm oil, distilled water, olive oil, lipoid- S 100, lecithin, thimerosal, and sorbitol	Antileukemic effect and acute toxicity	Acute toxicity of zerumbone and zerumbone-loaded nanocarriers via the oral route of administration	Both zerumbone and zerumbone-loaded lipid nanocarriers at acute doses do not induce behavioral alterations, toxicological signs, or adverse effects.
3	Nanostructured lipid nanocarriers [35]	Hot, high-pressure homogenization	**Lipid dispersion:** hydrogenated palm oil, olive oil, and lipoid S100 **Aqueous dispersion:** Sorbitol, Tween-80 and thiomersal	Colorectal adenocarcinoma	Increase the potency and efficacy of zerumbone-loaded lipid nanocarriers	Zerumbone lipid nanocarriers depict slow release of the drug without altering the anti-cancer effect.
4	Nanostructured lipid nanocarriers [28]	Hot, high-pressure homogenization	**Lipid dispersion:** hydrogenated palm oil, olive oil, and lipoid S100 **Aqueous dispersion:** Sorbitol, Tween-80 and thiomersal	Canine mammary gland tumor	Antiproliferative effect and the mode of cell death on canine mammary gland tumor adenocarcinoma	Effective in inhibiting the proliferation and inducing apoptosis on the canine mammary gland tumor cells. Inhibition of Bcl-2 and activation of pro-apoptotic Bax gene expressions and activation of caspases of the intrinsic and extrinsic apoptosis pathways were reasons for the effect.
5	Inclusion complexes [60]	Inclusion complex through freeze-drying	Hydroxypropyl-β-cyclodextrin	Anticancer drug effect	Enhancement of solubility of zerumbone	Important modifications in the solubility and stability of zerumbone
6	Nanosuspensions [48]	High-pressure homogenization	Hydroxypropyl methylcellulose (HPMC) and sodium dodecyl sulfate (SDS)	Not applicable	Improve solubility and dissolution characteristic’s	Enhanced dissolution and saturation solubility of zerumbone.
7	Nanostructured lipid carrier gel [47]	Hot, high-pressure homogenization	Carbopol 980 used to prepare the nanostructured lipid carrier gel	Wound healing	Anti-inflammatory activity	The gel decreased inflammatory cell infiltration and degeneration and increased granulation in healing wound tissues. There was an increase of anti-inflammatory IL-10, decreased the pro-inflammatory TNF-α, IL-6 concentrations, and downregulated cyclooxygenase-2 gene expression.

**Table 3:** Toxicity studies with zerumbone and zerumbone-based formulations.

Type of Study	Subjects	Dose	Key Results
Acute toxicity [31]	Sprague Dawley rats	100–3000 mg/kg	The results from this study showed that single injected doses of zerumbone at 100–200 mg/kg had no toxic effects on the renal and liver tissues of rats. The death of all experimental animals was reported at high doses of 2500 and 3000 mg/kg, and 20 and 40% of animals died at doses of 1500 and 2000 mg/kg respectively. In addition, a dose of 500 mg/kg induced nephrocellular and hepatocellular damage leading to renal and hepatic failure. The LD_50_ value was 1.84 g/kg when injected intraperitoneally.
Genotoxicity [59]	Chinese hamster ovary (CHO) cell lines	2.5 to 80 μM/mL	The results from this study reported that zerumbone at high concentrations had a genotoxic and cytotoxic effect on CHO cells. However, it failed to induced mutagenic effects on *Salmonella* *Typhimurium strain TA100*

**Table 4:** Patented systems related to zerumbone and zerumbone-based formulations.

Year	Patented System	Patent/Publication Number	Clinical Application
2019	A gel containing zerumbone from bitter ginger for curative treatment of diabetic ulcers	WO2019173890A1	A dermatological pharmaceutical composition in the form of a gel for topical use containing zerumbone.
2019	Method for treating an allergic disease	United States Patent 10688078	The invention relates to the use of zerumbone for treating an allergic disease.
2018	New use of zerumbone and compositions comprising zerumbone	EP2802310B1	The topical use of zerumbone for the treatment of the deficiencies of the capillary network of the skin. It also helps to treat micro-subcutaneous edemas, including bags and/or dark circles under the eyes.
2014	A composition for treating leukemia	WO2014123406A1	The composition comprises an effective amount of zerumbone and a pharmaceutically acceptable nanostructured lipid carrier for treating leukemia.
2013	Inclusion complex of zerumbone with a hydroxypropyl-β-cyclodextrin (*HP-β-CD*) in aqueous form	MY149711A	The present invention relates to a novel zerumbone inclusion complex having improved solubility properties.
2009	Immune modulation and anti-allergy activities of *Zingiber zerumbet*	United States Patent 7588788	The present invention provides for a method of preparing a nutraceutical formulation comprising zerumbone, and the use of this formulation to regulate the immune system, and more specifically to prevent or to treat an allergic disorder.
